# Gastric ESD under Heparin Replacement at High-Risk Patients of Thromboembolism Is Technically Feasible but Has a High Risk of Delayed Bleeding: Osaka University ESD Study Group

**DOI:** 10.1155/2013/365830

**Published:** 2013-06-13

**Authors:** Toshiyuki Yoshio, Tsutomu Nishida, Naoki Kawai, Kiyonori Yuguchi, Takuya Yamada, Takamasa Yabuta, Masato Komori, Shinjiro Yamaguchi, Shinji Kitamura, Hideki Iijima, Shusaku Tsutsui, Tomoki Michida, Eiji Mita, Masahiko Tsujii, Tetsuo Takehara

**Affiliations:** ^1^Department of Gastroenterology, Osaka National Hospital, 2-1-14 Hoenzaka, Chuo-ku, Osaka 540-0006, Japan; ^2^Department of Gastroenterology and Hepatology, Osaka University Graduate School of Medicine, Clinical Research Building (K1), 2-2 Yamadaoka, Suita, Osaka 565-0871, Japan; ^3^Department of Gastroenterology, Osaka Police Hospital, Kitayama-cho, Tennoji-ku, Osaka 543-0035, Japan; ^4^Department of Gastroenterology, Sakai City Hospital, 1-1-1 Minamiyasui-cho, Sakai-ku, Sakai 590-0064, Japan; ^5^Department of Gastroenterology, Osaka Rosai Hospital, 1179-3, Nagasone-cho, Kita-ku, Sakai 591-8025, Japan; ^6^Department of Gastroenterology, Itami City Hospital, 1-100 Koyaike, Itami-shi, Hyougo 664-8540, Japan; ^7^Department of Gastroenterology, Osaka Kousei-Nenkin Hospital, 4-2-78 Fukushima, Fukushima-ku, Osaka 553-0003, Japan

## Abstract

*Objectives*. Heparin replacement (HR) is often performed in patients with a high risk of thrombosis undergoing endoscopic procedures. However, information about the influence of HR is scarce. The aim of this study is to assess the clinical impact of HR for gastric endoscopic submucosal dissection (ESD). *Methods*. This is a retrospective study comprising approximately 1310 consecutive gastric neoplasms in 1250 patients, who underwent ESD in 5 institutes. We assessed the clinical findings and outcomes of ESD under HR, compared to ESD without HR as control. *Results*. A total of 24 EGC lesions in 24 patients were treated by ESD under HR. In the HR group, the complete en-bloc resection rate was 100%. The delayed bleeding rate was, however, higher in the HR group than in the controls (38% versus 4.6%). The timing of bleeding in the HR group was significantly later than in controls. In the control group, 209 patients discontinued antithrombotic therapy during perioperative period, and their delayed bleeding rate was not different from those without antithrombotic therapy (5.7% versus. 4.4%). A thromboembolic event was encountered in 1 patient under HR after delayed bleeding. *Conclusion*. ESD under HR is technically feasible but has a high risk of delayed bleeding.

## 1. Introduction

Endoscopic resection of early gastric cancer (EGC) started as endoscopic mucosal resection (EMR) [1] and has dramatically developed and been applied in many patients, owing to the establishment of criteria for node-negative tumors [2] and the advancements of endoscopic submucosal dissection (ESD) [3–6]. We recently reported, in a multicenter study, that ESD is a feasible method for treating EGC [7] and that long-term outcome of gastric ESD is satisfactory [8]. We also showed that almost all recurrent lesions, synchronous or metachronous, were treatable by endoscopic resection by scheduled endoscopic surveillance [8]. ESD has become a more acceptable option for EGC than gastrectomy in elderly patients, who often have several comorbidities [9] and accompanying medication such as antithrombogenic agents for the primary and secondary prevention of cerebrovascular and cardiovascular diseases. 

 Some patients with comorbidities such as valvular heart disease, atrial fibrillation with history of cerebrovascular accident have a high risk of developing thrombotic disease. Discontinuation of antithrombotic agents in these patients may cause life-threatening cerebrovascular and cardiovascular events. Such patients are often treated under heparin replacement (HR) of antithrombotic drugs, as a bridge therapy, to prevent thrombogenic events during therapeutic endoscopy or surgery. Patients requiring HR are increasing in Japan, where the elderly population is remarkably increasing; however, no reports on the clinical outcome and risk-benefit analysis of endoscopic resection under HR, including ESD, have been reported thus far.

 Here, we show the outcome and risk of gastric ESD under HR, especially with regard to delayed bleeding in a large multicenter study.

## 2. Methods

### 2.1. Patients

This is a retrospective multicenter study by Osaka University ESD Study Group. From March 2003 through February 2011, we consecutively treated gastric neoplasms with ESD at 5 institutes—Osaka National Hospital, Osaka University Hospital, Osaka Police Hospital, Sakai City Hospital, and Osaka Rosai Hospital. All indicated lesions were preoperatively confirmed to be adenocarcinomas or suspected adenocarcinomas through endoscopic biopsy. For patients with EGC receiving antithrombotic therapy and having a high risk of thrombosis, we performed HR as a bridge therapy during the perioperative period. Patients were divided into the HR group and the control group (without HR). 

### 2.2. ESD Procedure

 ESD was performed in hospitalized patients under moderate to deep sedation. ESD was principally indicated for possible node-negative EGCs according to the criteria of Gotoda et al. [2] based on endoscopic findings, including chromoendoscopy, endoscopic ultrasonography, and biopsy. Before ESD, written informed consent was obtained from each patient. The ESD procedure was performed as previously described [4, 6]. Each endoscopist used electrosurgical knives as necessary, such as IT knife (KD-610L or KD-611L; Olympus, Tokyo, Japan), Hook knife (KD-620LR; Olympus), Flex knife (KD-630L; Olympus), or Flush knife (DK-2618; FUJIFILM, Tokyo, Japan) and ICC200 (ERBE, Tübingen, Germany) or VIO 300D (ERBE) as an electrosurgical generator.

### 2.3. Management of Patients Taking Anticoagulants and Antiplatelet Agents

 When gastric ESD was scheduled in patients who are under treatment with oral anticoagulants such as warfarin and antiplatelet agents such as aspirin, ticlopidine, clopidogrel, and cilostazol, we consulted with the prescribing doctor before treatment. Patients who were judged by the prescribing doctor to have high-risk thromboembolism underwent HR, basically according to American Society for Gastrointestinal Endoscopy (ASGE) guidelines [10, 11]. Drug holidays before and after therapeutic endoscopy were determined according to the Japan Gastroenterological Endoscopy Society (JGES) guidelines: 3 days for aspirin and warfarin, 5 days for cilostazol, and 5–7 days for ticlopidine and clopidogrel before the procedure and reinitiated the next day after ESD. Unfractionated heparin (Heparin Sodium Injection “Ajinomoto”; Ajinomoto Pharmaceuticals Co., Ltd., Tokyo, Japan) was used for HR. Continuous administration of heparin was initiated and controlled to keep the activated partial thromboplastin time (aPTT) at approximately 60 seconds. Heparin was discontinued 4–6 hours before the ESD procedure. In some cases, the prothrombin time-international normalized ratio (PT-INR) was measured before ESD to confirm that the drug effect has disappeared. The next day after ESD, heparin sodium was readministered after confirming the absence of symptomatic gastrointestinal bleeding or a decrease in hemoglobin level. Warfarin was also administered the day after ESD when no evidence of bleeding was observed. Monitoring of PT-INR for warfarin and of the activated partial thromboplastin time for heparin was initiated after ESD. Heparin sodium was discontinued when the PT-INR level has reached approximately 1.50. In case of HR for antiplatelet agents, on the next day after ESD, antiplatelet agents were administrated simultaneously with heparin, and heparin was discontinued in three days.

### 2.4. Complications

Delayed bleeding has been defined as an event requiring emergency endoscopy with endoscopic hemostasis or transfusion for the management of hematemesis or melena and anemia of >2 g/dL decrease in hemoglobin level after ESD until a week after ESD. In Table 5 and Figure 2, in case endoscopic hemostasis was required 2 or 3 times because of rebleeding, we count each hemostasis procedure, though we did not count a case of anemia without clear event of bleeding. 

### 2.5. Statistical Analysis

All continuous variables are expressed as mean ± standard deviation (SD). Statistical analyses were conducted using the Student's *t*-test, Fisher's exact test, and chi-square test, and a probability of less than 5% was considered significant. All statistical analyses were performed with GraphPad PRISM (GraphPad Software, Inc.). 

## 3. Results

### 3.1. Clinical Background of ESD Patients

In 5 hospitals, 1310 consecutive EGC lesions in 1250 patients were subjected to gastric ESD. Among the total patients, 233 (18%) were taking warfarin or antiplatelet agents for their comorbidities, and 24 (1.9%) underwent HR before the procedure because of their high risk for thromboembolism. These 24 patients were placed in the HR group, and the other 1226 patients were categorized as the control group. Among the controls, 209 patients discontinued antithrombotic agents (antiplatelet, 187; anticoagulant, 39; both, 17) during the perioperative period because they were judged to be of low risk for thromboembolism. The remaining 1017 controls were not taking antithrombotic agents.

### 3.2. Clinical Findings in Cases of ESD under HR

Of the 24 patients with HR, 16 (64%) were taking warfarin, and 16 (64%) were taking antiplatelet agent, including 8 (32%) taking both of them. Aspirin (46%) was the most common antiplatelet agent, followed by ticlopidine (36%; Table 1). Patients were treated with 1.7 types of antithrombotic agents on average, and 62.5% of the patients were treated with multiple drugs. The most common comorbidity was atrial fibrillation (54.1%), followed by ischemic heart disease (45.8%), valvular heart disease (20.8%), and cerebral infarction (16.7%; Table 1).

The clinical findings of 24 patients in the HR group are shown in Table 2. Of them, 11 patients (37.5%) showed delayed bleeding. Among the resected lesions, the tumor size, tumor depth, ulceration (UL) positivity, and submucosal (SM) invasion frequency were not different between patients with or without delayed bleeding. The frequency of other comorbidities such as diabetes mellitus and chronic renal failure did not influence the rate of delayed bleeding. The number of anticoagulants or antiplatelet drugs received was significantly higher in patients with delayed bleeding (average, 2.1) than in those without bleeding (1.5, *P* < 0.05). 

### 3.3. Outcomes and Complications of ESD under HR Compared with Controls

 We also analyzed patients who underwent ESD without HR during the same period and in the same institutes as controls. The background of these patients and information about resected EGC lesions of the HR and control groups are shown in Table 3. Significantly more men and more upper-located lesions were included in the HR group than in the control group. In all patients treated with ESD under HR, en-bloc and R0 resection (resection with tumor-free lateral and basal margins) was achieved, and no additional surgical therapy was required for radical cure. 

**Figure 1:**
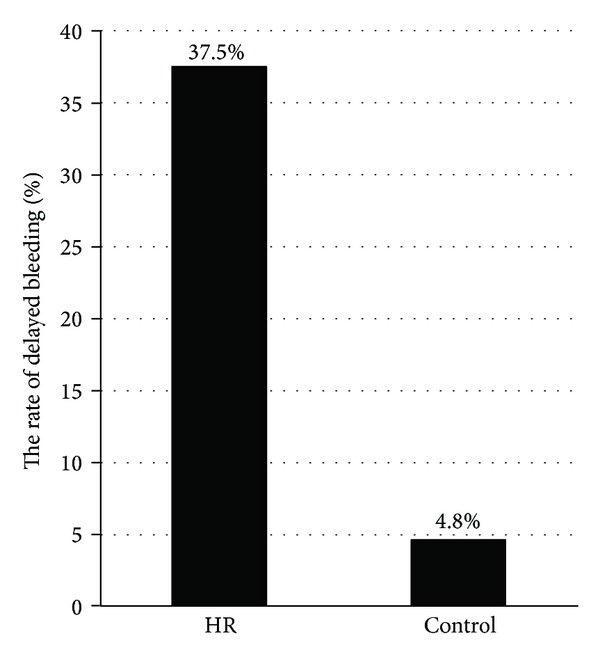
Comparison of the delayed bleeding rate between the ESD under HR group and the control group. Delayed bleeding occurred at a higher frequency in the ESD under HR group than in the control group (*P* < 0.0001).

The rate of delayed bleeding was significantly higher in patients who received ESD under HR (37.5%) than that in controls (4.8%) (*P* < 0.0001) (Table 4, Figure 1). Delayed bleeding occurred in 6 out of 16 patients who were taking anticoagulants with or without antiplatelet agents and 3 of 8 patients who were taking antiplatelet agents alone (Table 2). In the HR group, all bleeding events were successfully managed with endoscopic hemostasis; however, 2 patients (8.3%) required second sessions of endoscopic hemostasis because of rebleeding (Table 4). In the control group, 3 patients (0.2%) required second sessions of endoscopic hemostasis, 1 patient required an emergency operation, and others were managed by endoscopic hemostasis. Blood transfusion was performed in 3 patients (12.5%) in the HR group and in 11 patients (0.9%) in the control group (Table 4). 

 No perforation and other major complications related to ESD occurred in the HR group (Table 4). In the control group, 69 patients (5.4%) had perforation, and 4 patients (0.3%) required operation, including 2 patients with delayed perforations. 

Although the length of hospital stay according to the clinical pass of gastric ESD in all 5 institutes is approximately 7 days, the average length of hospital stay in the HR group was 22.5 days. This is because we required several days to control the anticoagulant effects before and after ESD in this group of patients. No thrombotic events were observed in controls; however, one patient (4.2%) with delayed bleeding in the HR group experienced a thrombotic event (Table 4).

This patient had been taken aspirin, ticlopidine, and cilostazol for ischemic heart disease and post cerebral infarction. He was performed ESD under HR. These 3 drugs were discontinued 7 days before ESD, and intravenous heparin started 4 days before ESD. ESD was successfully performed and then heparin restarted on the same day. After that we restarted all antiplatelet agents on postoperative day (POD) 1 and discontinued heparin on POD 4. He was discharged on POD 6, but he had delayed bleeding on POD 10. Emergently we performed endoscopic hemostasis and discontinued all antiplatelet agents. Although intravenous heparin was restarted on POD 11 and his aPTT was enough prolonged from 25.5 seconds to 45.8 seconds, cerebral infarction developed on POD 13.

### 3.4. Delayed Bleeding Occurred Later in the ESD under HR Group Compared with Controls

**Figure 2:**
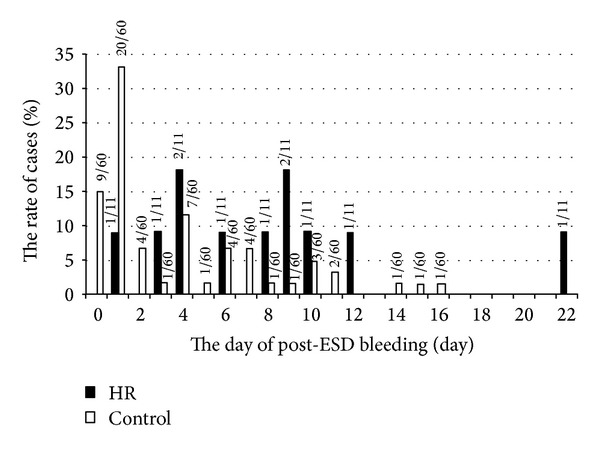
Comparison of the onset of delayed bleeding after ESD between the HR group and the control group. The days of delayed bleeding occurred after ESD in HR group (black bar) and control group (white bar) were shown in the graph.

 In patients who underwent ESD without HR, bleeding occurred on POD 3.8 ± 4.1, on average; however in the HR group bleeding occurred later (POD 8.0 ± 5.7) (Table 5, Figure 2). Between POD 0 and 1, half of all delayed bleeding events occurred in the control group and delayed bleeding rate was not so different between the HR group and controls (4.2% versus 2.4%). Between POD 2 and 22, bleeding occurred more frequently in the HR group than in controls (42% versus 2.5%) (*P* < 0.01) (Figure 2). 

### 3.5. Discontinuation of Antithrombotic Agents or Anticoagulants Prevented the Increase in Delayed Bleeding Rate

 Of the 1226 controls, 209 patients (17%) were using antithrombotic agents and were scheduled to discontinue these drugs during the perioperative period because of their low risk of thromboembolism. The delayed bleeding rate in these patients was 5.7% (12/209), which was almost similar to that of patients without antithrombotic agents (4.4%; 45/1017). Of the 209 patients with antithrombotic agents, 170 were using antiplatelet agents alone, 22 anticoagulants alone, and 17 both antiplatelet agents and anticoagulants, and the average number of antithrombotic agents was 1.2. The delayed bleeding rate did not increase in patients who discontinued taking antiplatelet agents (4.7%; 8/170) or in those who discontinued anticoagulants (4.5%; 1/22). In contrast, the delayed bleeding rate significantly increased in patients who discontinued both antiplatelet agents and anticoagulants (17.6%; 3/17), compared with those without antithrombotic agents (*P* < 0.05). However, these results were based on a small number of subjects.

## 4. Discussion

The number of patients receiving oral antithrombotic agents has been steeply increasing to prevent thromboembolic events in the last few decades. When patients were in high-risk conditions and taking anticoagulants such as warfarin in high-risk procedure like ESD, they need HR during the periendoscopic period. The ASGE guidelines, as well as the ESGE [12] and JGES guidelines, also recommend HR as a bridge therapy to reduce the risk of thromboembolic events [11]. 

Antiplatelet agents are commonly used in patients with coronary heart disease or peripheral or cerebral arterial stenosis. Although HR for anticoagulants is an accepted procedure as mentioned previously, a controversy still exists about whether HR should be used in patients taking antiplatelet agents. The recently published ESGE guidelines recommend that aspirin should be discontinued in high-risk procedures without HR [13]. A newly published Japanese guideline recommends that antiplatelet agents should be discontinued in patients with low risk of thromboembolism and in patients with high risk of thromboembolism and the procedures should be postponed, until patients can discontinue the antiplatelet agents. The Japanese guideline also suggests continuous use of antiplatelet agents in limitation of single use of aspirin (or cilostazol) in the perioperative period in high-risk procedure and does not mention anything about HR. However some cardiovascular and cerebrovascular doctors in Japan believe that HR may reduce the risk of thromboembolism in patients taking antiplatelet agents. Thus, HR is often performed clinically in Japan for the discontinuation of antiplatelets agents not only for the discontinuation of anticoagulants. This is why we used HR for some patients taking antiplatelet agents in this study. In a different background as Lee et al. reported that opinions and clinical practice patterns for the management of antithrombotic therapy differ between Eastern and Western endoscopists [13].

Until now, there have been few reports about the relation between antithrombotic therapy and delayed bleeding after ESD [9, 14]. Ono et al. showed appropriate perioperative cessation of antithrombotic agents did not increase the risk of delayed bleeding, but this report includes only a few patients with a high risk of thromboembolism who performed ESD under HR [14]. In this multicenter study, we also showed that appropriate cessation of antithrombotic agents prevented the increase in delayed bleeding in a large number of patients, when the condition risks of the patients allowed discontinuing these drugs. However, patients who were using both antiplatelet agents and anticoagulants were shown to have higher risk of delayed bleeding in this study, despite discontinuation of both agents. 

In this study, intraoperative bleeding was endoscopically well managed during gastric ESD under HR, resulting in high en-bloc resection rate and no requirement of emergency operation. ESD under HR is thus considered to be technically feasible. Generally, a large lesion size and middle- and lower-located lesion [15] have been reported as risk factors for bleeding. However, the delayed bleeding after ESD under HR occurred independently of tumor size and location (Table 2). In addition, the timing of delayed bleeding in ESD with HR was different from that without HR. In control group, the incidence of delayed bleeding on POD 0-1 (2.4%) was almost the same as that on POD 2–22 (Table 5), the same result as the previous report [16]. However, in the HR group the incidence of delayed bleeding on POD 2–22 was significantly higher than that on POD 0-1 (*P* = 0.017). These tendencies may result from the enhanced anticoagulant effect due to warfarin and heparin. In short, the clinical features of delayed bleeding after gastric ESD under HR include having high frequency, late onset, being recurrent, and being independent of lesion size and location. Further, to reduce delayed bleeding, intensifying post-ESD coagulation [15] and endoscopically closing the ulcer after ESD [17] may be considered, even though there is yet no evidence to prevent delayed bleeding in patients under HR. 

We experienced only 1 case of thrombotic event in a patient taking triple antiplatelets for ischemic heart disease and cerebral infarction, which occurred 3 days after delayed bleeding despite reinitiating heparin. In this case, intravascular volume depletion due to delayed bleeding may be one of the factors that caused the thrombotic event; hypercoagulability status after bleeding may be another cause. Therefore, preventing delayed bleeding is important to prevent thrombotic events in patients with a high risk for thromboembolism. As patients required HR for gastric ESD have substantial risk of thrombosis, we should pay enough attention to find early sign of thrombotic events and to start their treatments smoothly. 

Recently, Lim et al. reported that continuous administration of antiplatelet agents did not have an independent significant association with bleeding, although a higher bleeding rate was observed (no antiplatelets group, 5.2%; withdrawal group, 5.9%; continuous treatment group, 11.6%) [18]. In this study continuous treatment group is mentioned to include not only patients who had continued antiplatelet therapy but patients had it interrupted <7 days before ESD. So delayed bleeding rate can be a little bit higher in patients who continued antiplatelet agents throughout the perioperative period. But even so continuous administration of antiplatelet agents in high risk of thrombosis can be a good choice compared to HR during the ESD perioperative period. In the next study, we hope to evaluate in a prospective study whether continuous aspirin administration during the ESD perioperative period can prevent thrombogenic events in patients with high cardiovascular risks, in addition to not increasing the risk of delayed bleeding. 

This study has some limitations. First, this is a retrospective study. Second, because the EGC patients with indication of HR are not so many, although they are increasing, small sample size of HR group is the limitation of this study. 

In conclusion, gastric neoplasms could be satisfactorily resected by ESD under HR in patients at a high risk for thromboembolic diseases, although substantial risks of hemorrhagic and thromboembolic events remain. Such patients should be individually considered and given special attention to prevent the thromboembolic and bleeding risks related to the ESD procedure.

## Figures and Tables

**Table 1:** Summary of antithrombotic agents and comorbidities of patients treated with ESD under HR.

	Patients (%)
Antithrombotic agents	
Warfarin	16 (67%)
Aspirin	11 (46%)
Ticlopidine	9 (38%)
Clopidogrel	4 (17%)
Cilostazol	1 (4%)
Comorbidity	
Atrial fibrillation	13 (54.1%)
Ischemic heart disease	11 (45.8%)
Valvular heart disease	5 (20.8%)
Cerebral infarction	4 (16.7%)
Pacemaker	2 (8.3%)
Sick sinus syndrome	1 (4.2%)
Deep vein thrombus	1 (4.2%)
Pulmonary infarction	1 (4.2%)

**Table 2:** Profile and complications of EGC patients treated with ESD under HR.

Case	Delayed bleeding	Thrombogenic event	Age	Sex (M/F)	Tumor location (U/M/L)	Tumor size (mm)	Histological type	Depth of tumor	UL(−/+)	Comorbidities requiring anticoagulants or antiplatelet drugs	Anticoagulants or antiplatelet drugs	DM	CRF	Other comorbidities
1	−	−	62	M	M	23	tub1	M	−	Af, valvular heart disease	Warfarin, ticlopidine	−	−	
2	−	−	63	M	M	20	tub1	M	−	Af, valvular heart disease	Warfarin, ticlopidine	−	−	
3	−	−	76	M	U	25	tub1	SM1	−	IHD (DES)	Aspirin, ticlopidine	−	−	Asthma
4	−	−	71	M	L	31	tub1	M	−	Af	Warfarin	−	−	
5	−	−	74	M	U	16	tub1	M	−	Af, cerebral infarction	Warfarin	−	−	
6	−	−	65	M	L	4	tub1	M	−	Af, cerebral infarction	Warfarin	+	−	
7	−	−	65	M	U	10	tub1	M	−	Pulmonary infarction, deep vein thrombosis	Warfarin	−	−	
8	−	−	73	M	M	14	tub1	M	−	IHD	Aspirin, clopidogrel	−	+	History of tuberculosis
9	−	−	80	M	L	6	tub1	M	−	Cerebral infarction	Clopidogrel	−	−	Colon cancer after operation, myasthenia gravis
10	−	−	70	M	U	20	tub1	M	−	Af, IHD	Warfarin	−	−	
11	−	−	82	M	L	6	tub1	M	−	Af	Warfarin	+	−	
12	−	−	60	M	L	9	tub1	M	−	IHD (DES)	Aspirin, ticlopidine	−	−	Hypertension, hyperlipidemia
13	−	−	63	M	M	14	tub1	SM1	−	Af	Warfarin	+	−	
14	−	−	78	M	U	40	tub1	M	−	IHD	Aspirin, clopidogrel	+	−	
15	−	−	71	M	U	30	tub2 > por	M	+	Af, IHD (DES)	Warfarin, aspirin	+	+	
16	+	−	63	M	M	16	tub1	M	−	Af, valvular heart disease	Warfarin, ticlopidine	−	−	
17	+	−	63	M	M	32	tub2 > tub1	M	−	Af, valvular heart disease	Warfarin, ticlopidine	−	−	
18	+	−	64	M	L	12	tub1	M	−	IHD, valvular heart disease	Warfarin, aspirin	−	−	
19	+	−	66	M	M	28	tub1	M	−	SSS with pacemaker	Warfarin	−	−	
20	+	−	73	M	L	23	tub1	M	−	Af, valvular heart disease	Warfarin, aspirin, ticlopidine	−	−	Hypertension
21	+	−	68	M	M	10	tub1 > tub2	M	−	Af, IHD with pacemaker	Warfarin, aspirin	−	−	
22	+	−	62	M	U	14	tub1	M	−	IHD (DES)	Aspirin, clopidogrel	+	−	Chronic hepatitis type C
23	+	−	57	M	M	8	tub1	M	−	IHD	Aspirin, ticlopidine	−	−	
24	+	+	71	M	U	15	tub1	M	−	IHD, cerebral infarction	Aspirin, ticlopidine, cilostazol	+	−	Hypertension, hyperlipidemia

Abbreviations; Af: Atrial fibrillation; IHD: Ischemic Heart Disease; DES: Drug Eluting Stent; SSS: Sick Sinus Syndrome.

**Table 3:** Clinicopathological findings of patients and lesions treated with ESD with or without HR (control).

Patients	HR (*n* = 24)	Control (*n* = 1226)	*P* value
Age (y)	68 ± 7	70 ± 8.6	0.26
Sex (M/F)	24/0	927/299	<0.01
Number of resected lesions (1/2/3<)	24/0/0	1167/53/6	0.55

Lesions	HR (*n* = 24)	Control (*n* = 1286)	*P* value

Location (U/M/L)	8/9/7	171/419/696	<0.01
Lesion size (mm)	17.8 ± 9.3	16.8 ± 11.6	0.68
Specimen size (mm)	36.3 ± 9.9	37.0 ± 12.5	0.82
Pathological findings (pap/tub1/tub2/por/sig/adenoma/others)	0/22/2/0/0/0/0	3/913/133/2/4/190/41	0.62
Depth of lesion (M/SM1/SM2<)	22/2/0	942/55/58	0.41
UL (−/+)	21/1	854/88	0.44

**Table 4:** Summary of endoscopic outcomes and complications associated with ESD under HR.

Patients	HR (*n* = 24)	Control (*n* = 1226)	*P* value
Delayed bleeding	9 (37.5%)	57 (4.8%)	<0.01
Transfusion	3 (12.5%)	11 (0.9%)	<0.01
Multiple sessions of hemostasis	2 (8.3%)	3 (0.2%)	<0.01
Thrombogenic event	1 (4.2%)	0	<0.05

Lesions	HR (*n* = 24)	Control (*n* = 1286)	*P* value

En-bloc and R0 resection	24 (100%)	1249 (97.1%)	1
Perforation	0 (0%)	69 (5.4%)	0.633

**Table 5:** Delayed bleeding after ESD with or without HR.

	HR (*n* = 24)	Control (*n* = 1226)	*P* value
Delayed bleeding	9 cases 11 times	57 cases 60 times	
Average period of delayed bleeding (days)	8.0 ± 5.7	3.8 ± 4.1	<0.01
